# Algorithms in the court: does it matter which part of the judicial decision-making is automated?

**DOI:** 10.1007/s10506-022-09343-6

**Published:** 2023-01-08

**Authors:** Dovilė Barysė, Roee Sarel

**Affiliations:** 1 Institute of Psychology, Vilnius University, University Str. 9, 01513 Vilnius, Lithuania; 2 Institute of Law and Economics, University of Hamburg, Johnsallee 35, 20148 Hamburg, Germany

**Keywords:** Judicial decision-making, Human-automation interaction, Legal technologies, Levels of automation, Perceived fairness

## Abstract

Artificial intelligence plays an increasingly important role in legal disputes, influencing not only the reality outside the court but also the judicial decision-making process itself. While it is clear why judges may generally benefit from technology as a tool for reducing effort costs or increasing accuracy, the presence of technology in the judicial process may also affect the public perception of the courts. In particular, if individuals are averse to adjudication that involves a high degree of automation, particularly given fairness concerns, then judicial technology may yield lower benefits than expected. However, the degree of aversion may well depend on how technology is used, i.e., on the timing and strength of judicial reliance on algorithms. Using an exploratory survey, we investigate whether the stage in which judges turn to algorithms for assistance matters for individual beliefs about the fairness of case outcomes. Specifically, we elicit beliefs about the use of algorithms in four different stages of adjudication: (i) information acquisition, (ii) information analysis, (iii) decision selection, and (iv) decision implementation. Our analysis indicates that individuals generally perceive the use of algorithms as fairer in the information acquisition stage than in other stages. However, individuals with a legal profession also perceive automation in the decision implementation stage as less fair compared to other individuals. Our findings, hence, suggest that individuals do care about how and when algorithms are used in the courts.

## Introduction 

Artificial Intelligence (“AI”) is swiftly becoming a relevant component in judicial decision-making processes around the globe (see, e.g., Reiling 2020). In China, “internet courts” already provide an online dispute resolution mechanism, also involving AI components (Fang 2018; Shi et al. 2021). In the US state of Wisconsin, judges utilize algorithms to derive recommended criminal sentences (Beriain 2018). Assessments of the defendant’s risk of engaging in violent acts are increasingly used in many countries (Singh et al. 2014) with varying degrees of accuracy (Tolan et al. 2019; Greenstein 2021).^1^ Such technologies are typically referred to as Algorithmic Decision Making (“ADM”; see, e.g., Newell and Marabelli 2015; Araujo et al. 2020).^2^

Straightforwardly, judges can benefit from the presence of ADM in judicial proceedings (Reichman et al. 2020; Winmill 2020).^3^ First, advanced automation can potentially reduce the effort cost required to search through the documents, seek out the relevant legal provisions, or apply the law to the facts of the case.^4^ Second, the well-established benefit of ADM lies in its ability to provide predictions that humans find difficult to generate, e.g., because the human capacity required to detect patterns in complex cases is limited (Alarie et al. 2018; De Mulder et al. 2022). Hence, ADM can potentially increase judicial accuracy by providing new information that cannot be detected by the naked eye or by improving the analysis process.

Nevertheless, it is currently somewhat difficult to study how ADM affects judicial decision-making, as the inner-working of judicial decision-making can be opaque, so it is not easy to observe which technology has been used. More importantly, the use of technology is not binary. Instead, judges may turn to ADM in different stages of adjudication, albeit the exact features vary across judicial systems. For instance, a highly technological court may take the following form: in the early stages of the judicial process, parties may be asked to upload their statements (e.g., the lawsuit and the statement of defense) onto a computerized system, while entering some general details about the lawsuit (sum, type of lawsuit, details on the parties). The judge can then observe the documents and verify whether they are consistent with the relevant procedural rules. After the initial submission, judges can use a computerized system to keep track of the process, send out automated reminders on deadlines, or run any number of preliminary analyses. Algorithms can then play a continuous rule in the adjudication process, e.g., by generating predictions, providing assessments and issuing electronic decisions. The more the technology develops, additional automated functions might be added to such a process. In the extreme case, a digitized dispute resolution might eventually be used to replace the judge and thereby circumvent the courts altogether (see, e.g., Ortolani 2019). However, we will restrict attention to a human judge who delegates some (but not all) of the judicial functions to ADM.

There have been extensive discussions on the implications of using automated procedures in the judicial process. These include aspects such as transparency and accountability, judicial independence, equality before the law, diversity, right to a fair trial, and efficiency (Matacic 2018; Zalnieriute and Bell 2019; Morison and Harkens 2019; Wang 2020; English et al. 2021). In particular, a salient concern for using technologies by judges is unfairness, e.g., algorithms can generate discriminatory outcomes based on race, ethnicity, or age (e.g., Jordan and Bowman 2022; Köchling and Wehner 2020). Moreover, some studies suggest that judges might use technology selectively, as they tend to rely more on extralegal factors in severe cases (Cassidy and Rydberg 2020).^5^ At the same time, existing studies from behavioral economics suggest that judges may be susceptible to various cognitive biases (e.g., Guthrie et al. 2000, 2007; Winter 2020),^6^ raising the question of whether the use of ADM can de-bias judges (see, e.g., Chen 2019).

Nonetheless, the concern of unfairness may well harm public trust in the judicial system. As one recent example, the use of software known as “COMPAS” to assess individual risk of reoffending has led to public outrage following the discovery that the algorithm led to racially discriminatory outcomes (Zhang and Han 2022).^7^ Another famous example includes the fraud detection system “SyRI” in the Netherlands, which collected large amounts of personal data. SyRI was challenged by civil rights organizations in the District Court of The Hague, which then ruled that the technology violates the right to privacy (van Bekkum and Borgesius 2021; Buijsman and Veluwenkamp 2022).

These examples could be viewed as a special case of a more general issue: the relationship between AI and trust. In a recent review, Glikson and Woolley (2020) survey over 200 papers published in the last 20 years and identify different dimensions that determine whether individuals trust AI,^8^ such as tangibility, transparency, reliability, and the tasks’ degree of the tasks’ technicality. These determinants are then found to have different effects, depending on how the AI manifests itself (as a physical robot, a virtual agent, or an embedded component). For instance, low reliability seems to decrease trust when the AI is embedded but may or may not decrease trust when the AI manifests itself as a robot. Such complexity makes it difficult to speculate on how judges respond to advice generated by ADM and, by extension, how this affects the public’s trust in those judges.^9^

There is extensive writing on the importance of public trust in the courts (see., e.g., Burke and Leben 2007; Gutmann et al. 2022; Jamieson and Hennessy 2006), on the one hand, and the importance of trust in technology (Madhavan and Wiegmann 2007; Lee 2018; da Silva et al. 2018; Felzmann et al. 2019), on the other hand. However, the intersection is (at least empirically) under-explored,^10^ with a few exceptions. Hermstrüwer and Langenbach (2022) use a vignette study to elicit perceptions of fairness in three contexts (predictive policing, school admissions, and refugees) on a scale ranging from “fully human” to “fully automated”. They find that purely algorithmic analysis is considered the least fair, but purely human decision-making is also considered somewhat unfair. Conversely, their study finds that combining automated processes with high human involvement yields a higher fairness perception. Yalcin et al. (2022) conducted a vignette experiment over MTurk and found that subjects care whether the judge is a human or an algorithm, finding evidence of higher trust in human judges.

Our study asks a related question: do individuals care about *the stage* in which technology is used by judges (rather than the overall degree of automation)? This question is crucial because it allows refining the conclusion as to what individuals (dis)like about the combination of human and machine adjudication. Specifically, we utilize a taxonomy by Parasuraman et al. (2000) that differentiates between four different stages of decision-making: (i) information acquisition, (ii) information analysis, (iii) decision selection, and (iv) decision implementation.

For each of these, we elicit beliefs about the Level of Automation (“LOA”) most likely to ensure the fairest outcome using an online exploratory survey of 296 participants. Our analysis yields two main findings. *First*, we find that individuals believe that an intermediate LOA generates the fairest results in the information acquisition stage, which is consistent with the study by Hermstrüwer and Langenbach (2022). However, we also find that lower levels of automation are believed to generate fairer outcomes in the remaining stages. This result suggests that individuals' preferences for combining human decision-making and algorithms is driven by AI’s relative advantage in *acquiring information* rather than its advantage in analyzing it. In other words, people trust judges to apply their legal expertise but less so to gather the relevant information. This conclusion seems particularly relevant for the distinction between inquisitorial and adversarial systems, as the judge's role in evidence collection is more passive in the latter than in the former.

*Second,* we find evidence that individuals with a legal profession believe that lower levels of automation are fairer in the *implementation stage.* This suggests that lawyers, unlike laypeople, are even more skeptical toward the AI’s ability to execute judicial decisions, i.e., lawyers tend to trust judges more strongly when it comes to implementation.^11^

Our results seem important both for institutional design (e.g., how much technology to allow in judicial decision-making) and for judges who operate within those institutions.

The remainder of the paper is organized as follows: Sect. 2 situates our study within the existing literature. Section 3 describes our study’s design, with results reported in Sect. 4. Section 5 discusses the results, highlights some limitations, and concludes.

## Related literature

Our paper is related to several streams of literature, including existing attempts to classify legal technologies, perceived procedural fairness of algorithms (in particular, in judicial decision-making), and work on the relative advantage of technology versus humans in judicial processes. We summarize the relevant points in turn.

### A taxonomy of judicial decision-making automation

There are several existing attempts to create some classification for legal technologies. The Stanford University Codex Techindex^12^ (see, e.g., McMaster 2019) categorizes existing legal technologies into nine categories^13^ but does not discern between technologies intended for laypeople and for experts. These categories also do not easily lend themselves to researching decision-making or automation. A different attempt is contained in a report by “The Engine Room” (Walker and Verhaert 2019), which focuses on legal-empowerment technologies. Unfortunately, this attempt mainly revolves around technological applications and does not strive to provide a comprehensive categorization. The Law Society of England and Wales launched another attempt (Sandefur 2019), distinguishing between two “waves of AI in law”: a first (“rules-based”) wave, comprised of document automation, legal diagnostics, and legislative analysis tools, and a second wave, which embodies attempts to predict outcomes of disputes, analyze documents, and perform risk assessments. Much like the others, this attempt aims to describe current technological solutions rather than provide a clear taxonomy. More recently, Whalen (2022) proposed to classify legal technologies according to their “legal directness” and “legal specificity”,^14^ whereas Guitton et al. (2022a, b) suggest mapping regulatory technologies along three different dimensions^15^: the project’s aim, divergence of interests between stakeholders, and the degree of human mediation. A different approach was taken by Tamò-Larrieux et al. (2022), who propose the concept of Machine Capacity of Judgment (MCOJ). According to this concept, classification should be derived by the artificial agent’s autonomy (i.e. freedom from outside influence),^16^ decision-making abilities (including understanding the impact of decisions and balancing different options), and degree of rationality. Tamò-Larrieux et al. (2022) propose assigning a score to these parameters and leveraging those scores to determine how much to rely on the AI in question.

While these recent proposals seem useful for identifying what constitutes LegalTech and who it influences, they are less suitable for capturing how and when the technology is used by judges in their decision-making process.

We, therefore, take a different approach: combining elements from existing taxonomies and adjusting them to classify legal technologies. The starting point follows Parasuraman et al. (2000),^17^ which break down the process of decision-making into four stages: (i) *information acquisition*—gathering, filtering, prioritizing, and understanding the data; (ii) *information analysis*—analyzing, interpreting, and making inferences and predictions; (iii) *decision selection*—prioritizing/ranking decision alternatives; and, (iv) *decision implementati*on—executing the choice (e.g., writing-up and submitting the relevant document). While the categorization is not specific to legal decisions, it applies to judicial decision-making. For example, to make a ruling in the case, a judge must acquire relevant case law, analyze the information, generate alternatives, choose the best one, and implement the decision (e.g., write up a verdict). Notably, the stages are mutually inclusive, as to select the most relevant argument, one has to identify it and analyze it. Nevertheless, this taxonomy seems helpful for analyzing legal decision-making (see, e.g., Petkevičiūtė-Barysienė 2021).

This decision-making categorization is also closely related also to a paper by Proud et al. (2003), which describes the stages slightly differently (as “observe”, “orient”, “decide”, and “act”), but is nonetheless helpful for our purposes. They propose an LOA scale along the four stages of decision-making, with the underlying assumption that the preference may differ for each stage. We follow this assumption and elicit the beliefs of our survey respondents on fairness generated by the LOA in each stage of the judicial decision-making progress on a 5-point (Likert) scale ranging from “Manual” (i.e., no automation) to “full” (fully automated).^18^ We describe the precise definitions in Table 1.

**Table 1:** Taxonomy of levels of automation and decision-making stages

Decision-making stage	Level of automation			
	Low	Intermediate	High	Full
Information acquisition (A)	The computer gathers, displays and offers filtering	The computer gathers, displays and offers filtering as well as highlights relevant information for the human	The computer gathers, filters, and prioritizes information displayed to the human	The computer gathers, filters, and prioritizes data without displaying any information
Information analysis (B)	The computer analyzes the data and makes predictions, though the human is responsible for interpretation of the data	The computer overlays predictions with analysis and interprets the data. The human shadows the interpretation for contingencies	The computer analyzes, predicts, interprets, and integrates data into a result which is only displayed to the human if result fits programmed context (context dependent summaries)	The computer predicts, interprets, and integrates data into a result which is not displayed to the human
Decision selection (C)	Both human and computer perform ranking tasks, results from the human are considered prime	The computer performs ranking tasks. All results, including "why" decisions were made, are displayed to the human	The computer performs ranking tasks. The computer performs final ranking and displays a reduced set of ranked options without displaying "why" decisions were made	The computer performs ranking tasks. The computer performs final ranking, but does not display results
Decision implementation (D)	The computer executes the decision after human approval	The computer allows the human a context-dependent restricted time to veto before execution. Human shadows for contingencies	The computer executes automatically and only informs the human if required by context. It allows for an override ability after execution. The human shadows for contingencies	The computer executes automatically and does not allow any human interaction

This table presents the taxonomy used in our survey. For brevity, we omit the lowest level (“Manual”) as it simply means no automation, so that no further description is needed

### Perceived (procedural) fairness of ADM

Procedural fairness has long been applauded as means to keep litigants satisfied, cooperative, and trusting in the courts (see, e.g., Burke 2020; Burke and Leben 2007; MacCoun 2005). The presence of ADM in court procedures may, intuitively, affect both procedural fairness and the public *perception* of fairness. Studies on the perception of procedural fairness when AI is involved (not necessarily in the context of courts; see, e.g., Woodruff et al. 2018; Lee 2018; Lee et al. 2019; Saxena et al. 2019)^19^ yield some mixed results. Some studies find that algorithms are seen as less fair than humans (Newman et al. 2020; Hobson et al. 2021), e.g., because they lack intuition and subjective judgment capabilities. Other studies, however, find that that the difference in perceived procedural fairness of human decision-makers versus algorithmic decision-makers is task-dependent (e.g., Lee 2018).

In the specific context of judicial decision making, recent studies show that people tend to trust human judges more than algorithms (see Yalcin et al. 2022) or that they, at least, do not trust a fully-automated judicial process (see Hermstrüwer and Langenbach 2022). Kim and Phillips (2021) further argue that “a robot would need to earn its legitimacy as a moral regulator by demonstrating its capacities to make fair decisions”.^20^

Legal scholars seem to be divided on their attitude toward the use of technology in the course. While some seem supportive of such technologies (e.g., Reiling 2020; Winmill 2020), others take a more conservative approach (see, e.g., Sourdin and Cornes 2018; Ulenaers 2020). Often expressing concern of algorithmic bias (for a recent discussion, see Kim 2022). Such conservatism seems consistent with procedural fairness concerns, but may also driven by other reasons, e.g. dissatisfactory experience with technologies (Barak 2021), a fear of becoming redundant (Sourdin 2022), concern of being pressured into using more technologies (Brooks et al. 2020), or simply “Automation Bias” (Cofone 2021).

### Relative advantage, compatibility, and personal innovativeness in information technology

Individuals might prefer a different LOA due to various reasons, including their attitudes towards technology. However, the existing literature on the determinants of technology acceptance suggests that these depend on social context and are subject to heterogeneity. For example, “innovators”, as Rogers calls them (Rogers 2003; for a summary of the theory, see, e.g., Kaminski 2011), are willing to take risks, are the first to develop new ideas, and are easy to persuade to accept new technologies. Other groups follow different patterns. “Laggards”, for example, will remain conservative and skeptical even after the implementation of the technology. Most people, however, fall into two other (and more moderate) groups—“early majority” and “late majority”. The early majority demands evidence about the usefulness of the technology before they are willing to adopt it. The late majority needs more than that—they demand information on the technology’s success among other people. Accordingly, personal innovativeness in information technology is an often-used construct in the context of technology acceptance (see Ciftci et al. 2021; Patil et al. 2020; Turan et al. 2015).

These studies identify several factors that influence whether a person will belong to a group that is quicker to accept technology. We focus on two such factors—Relative Advantage and Compatibility—which seem the most closely related to judicial decision-making.^21^ The first factor, *Relative Advantage,* refers to the degree to which an innovation is seen as better than the idea, program, or product it replaces. The second factor, *Compatibility,* refers to whether the technology is consistent with the potential adopters' values, experiences, and needs. In other words, the first factor deals with whether the technology used by judges is a proper substitute for human decision-making, whereas the second factor deals with personal preferences.

Our study controls for these factors, along a few others (e.g., general trust and knowledge in legal technology) in order to isolate the question of interest—whether or not individuals care about the stage in which the judge turns to automation for assistance.

## Study design

The following sections describe our study design. Section 3.1 describes our participants. Section 3.2 explains the method and procedure.

### Recruitment of participants

We designed an online survey to elicit people’s beliefs about the fairness of using varying LOA for the different stages of the judicial process. Lithuania was chosen for the study,^22^ for two main reasons. First, Lithuania has relatively average legal technologies: there is no AI used directly by judges in courts but there is a history (long before COVID-19) of using technology generally in the courts, e.g. e-services portal for courts,^23^ judicial information systems,^24^ and audio recordings of court hearings (Bartkus 2021).^25^ The generally increasing level of automation in Lithuania is beneficial, as it makes it more likely that individuals will have varying degrees of awareness of at least some automation in courts. Second, Lithuania was chosen also for reasons of convenience, given that we had a logistical comparative advantage, which allowed us to recruit participants from several relevant groups (e.g., lawyers and other people with court experience) in this country with greater ease.

Participants were invited to participate using several recruitment methods. Given the different levels of knowledge and experience within legal systems, we wanted to recruit people both within and outside of the legal profession, and both with and without court experience (e.g., litigants, observers, defense attorneys, judges). Individuals from the legal community were invited to participate in the study by posting an invitation to a popular legal news site,^26^ emailing scholars from several universities,^27^and “snowballing”, i.e., reaching out to attorneys, judges, and legal scholars and asking them to share the invitation to participate with their colleagues. The snowballing sampling was also used to recruit people who have been to courts in any role, e.g., litigant, judge, observer, as court experience may greatly influence how people comprehend court work. The non-lawyer portion of the sample was approached by posting in various other Facebook groups.^28^ In order to reach a wider range of ages among participants, emails with invitations to participate in the study were shared with elderly people attending Medard Čobot’s Third Century University (distributed by the university’s administration).^29^ Given the variety of methods used, we cannot guarantee that our sample is representative of the entire population in Lithuania (or of the legal community in Lithuania). To mitigate this issue, we added several control variables (see the following section), which allow us to account for the heterogeneity among the participants. Overall, a convenience sample of 269 Lithuanian respondents participated in the study from May to June of 2021.

### Method and procedure

The survey consists of several steps. First, we elicited information used to generate control variables. These include some demographics (age and gender) but, more importantly, measures for specific attributes that may influence the respondents’ attitude toward the use of ADM in the legal sphere. Specifically, respondents were presented with sets of 7 statements about various legal technologies in courts alongside Likert scales to measure the relevant feature (for the sources used to derive the statements, see Table 9 in Appendix B)^30^:

- *Knowledge about Legal Technologie****s*** (“Knowledge in Tech”)—statements concerning the respondent’s general knowledge of legal technology. For instance, one statement was “In some countries, judges have access to a program that provides the judge with a detailed analysis of the case, evaluates arguments, and identifies possible outcomes of the case”. Then they are asked to indicate their *level of knowledge* on a Likert scale from 1 (“I know absolutely nothing about this”) to 5 (“I have tried this or a similar technology”).^31^
- *Trust in legal technologies technology* (“Trust in Legal Tech”), a scale consisting of three revised/adopted items, e.g., “Overall, I could trust legal technologies in courts”.
- *Relative advantage of legal technologies in courts* (“Relative Advantage”)—a scale consisting of five revised/adopted items, e.g., “I think legal technologies would help save time for court clients and staff compared to how courts operate now”.
- *Compatibility*—a scale consisting of four revised/adopted items from existing papers, e.g., “I think legal technologies would be well in line with my beliefs about how courts should operate”.
- *Personal innovativeness in information technology* (“Personal Innovativeness”)—a scale consisting of four revised/adopted items, e.g., “Among my peers, I am usually the first to explore new information technologies”).^32^

We pooled each set of statements using a simple mean, so that each feature is captured by one variable in the analysis.

The second stage of the survey involved the elicitation of the LOAs, i.e., the belief about which level of automation in the four stages (information acquisition, information analysis, decision selection, and decision implementation) is most likely to generate the fairest outcome. Respondents first read a few general sentences (e.g., “judges need a wide range of information to decide a case—legislation, decisions in similar cases, and legal arguments. The alternatives for outcome and arguments depend on the information found.”). Then, they were asked to “*Choose the option you think would best ensure the fairest verdict in most cases.*” As mentioned above, the answer was elicited on a 5-point Likert scale, ranging from a “Manual" to a “Full” automation level.^33^ This was done with each of the four stages separately—i.e., each decision-making stage was described on a separate page, and the participant chose a level of automation for each decision making-stage separately. Tables 9 and 10 in Appendix B provide a complete translation of the questions given to the respondents.

### Descriptive statistics

Descriptive statistics for our independent variables are presented in Table 2 (see also Table 4 in Appendix A for bivariate correlations). The sample has a mean age of approximately 41 years but also includes younger and older participants, which is essential given the possible generational gap regarding technology acceptance. There are slightly more females (60.7%) in the sample, and there are both participants with and without court experience.^34^ Given the importance of awareness about how courts operate, our sample contains a substantial number of people with court experience: over 50 percent of the participants (N = 143, see Table 2) had court experience with a variety of roles during the proceedings, i.e., 16 people observed the process, 38 were witnesses, 23-legal representatives, 24 litigants, 5 experts, 4 defendants in criminal proceedings, 13 victims of a crime, 5 prosecutors and 2 judges.

**Table 2:** Descriptive Statistics

Variable	Obs	Mean	Std. dev.	Min	Max
Age	256	41.281	12.381	23	81
Female	262	.607	.489	0	1
Legal profession	266	.199	.4	0	1
Court experience	268	.534	.5	0	1
Knowledge in legal tech	269	1.756	.817	1	5
Trust in legal tech	269	4.696	1.37	1	7
Relative advantage	269	5.085	1.243	1	7
Personal innovativeness	268	4.769	1.352	1.25	7
Compatibility	269	4.812	1.352	1	7

This table presents descriptive statistics for the independent variables used in the analysis

Respondents reported relatively low levels of knowledge in legal technologies but rather high trust in such technologies. Furthermore, the descriptive statistics show a relatively strong belief in the relative advantage of legal technologies.

Table 5 in Appendix A compares the descriptive statistics between those with and without a legal profession, showing that lawyers in our sample have more knowledge about legal technologies (*p* < 0.001), but do not differ on trust, personal innovativeness, or compatibility.

## Results

### Perceived fairness generated by levels of automation

**Fig. 1:**
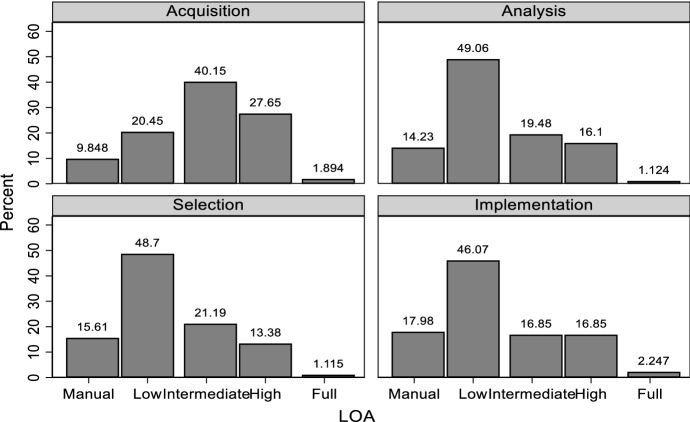
Perceived Fairness for each Level of Automation by stage of judicial decision-making

We begin our analysis by presenting descriptive results of LOA compared across the four stages of the judicial process in our taxonomy. The descriptive results are presented in Fig. 1 (a more detailed version is provided as Table 6 in Appendix A). The figure displays the percentage of responders who indicated that a particular LOA is most likely to produce a fair outcome.^35^ The figure clearly shows that the density of subjects choosing an intermediate level is high in the information acquisition stage (40.15%), but lower in other stages (ranging from 16.85 to 21.19%). Respectively, the density of respondents choosing “low” is the highest in the other stages.

A Person chi-squared test reveals that LOAs differ across the stages (*p* < 0.001). As we elicited multiple LOAs from each participant, we also checked for within-subject differences using a Repeated Measures ANOVA, confirming that there are statistically significant differences between the beliefs regarding fairness generated by LOAs for different stages of decision-making (*p* < 0.001). Overall, this check reveals two key insights. First, the LOA for the information acquisition stage differs from the LOA for all other decision-making stages (*p* < 0.001 in all cases). Second, the LOAs for the other three stages do not differ from each other (*p* > 0.05 in all cases).

**Fig. 2:**
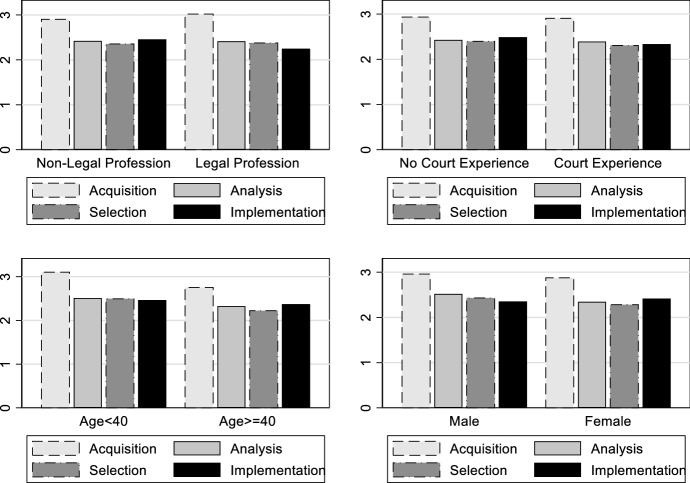
LOA by different characteristics

Next, Fig. 2 breaks down the data by different characteristics: Age (comparing older and younger respondents), gender, legal profession, and court experience (exact numbers are provided in Table 7 in Appendix A). The figure demonstrates that LOAs seem to be quite similar across different characteristics on a descriptive level (the only significant differences are between age groups). However, this does not yet constitute a full-blown analysis, as the variables capturing the characteristics are correlated (see Table 4 in Appendix A, which presents bivariate correlations). Hence, we proceed by using a regression model and control for these features simultaneously.

### Linear regressions

Linear regressions (OLS) were conducted to predict preferred levels of automation in judicial decision-making stages. The regression model is

$$LOA = \beta_{0} + \beta_{1} Analysis + \beta_{2} Selection + \beta_{3} Implementation + \beta_{4}^{\prime } X + \epsilon ,$$

where the first three variables are dummies for the stage of the judicial decision-making (so that *information acquisition* is the baseline category), X is a vector of varying controls, and $\epsilon$ is the error term. Results are reported in Table 3 (a full table, including the coefficients of the controls, is provided as Table 8 in Appendix A). As each observation represents one decision of one respondent (each respondent provided an answer for the four stages, so that there is a maximum of 1076 observations),^36^ we cluster the standard errors by respondent. Column (1) excludes controls. Column (2) adds demographics (gender, age, age-squared, legal profession, court experience). Column (3) adds the elicited Knowledge in LegalTech, Trust in LegalTech, Personal Innovativeness, and Compatibility. Column (4) adds an interaction term between the stages and legal profession. Column (5) replaces the controls with respondent fixed effects, in order to control for any feature that varies by subject but is, for whatever reason, unaccounted for by our controls.

**Table 3:** OLS Results

	(1)	(2)	(3)	(4)	(5)
Analysis	− 0.50*** (0.06)	− 0.49*** (0.07)	− 0.49*** (0.07)	− 0.48*** (0.08)	− 0.48*** (0.08)
Selection	− 0.56*** (0.06)	− 0.54*** (0.07)	− 0.54*** (0.07)	− 0.52*** (0.08)	− 0.52*** (0.08)
Implementation	− 0.52*** (0.07)	− 0.53*** (0.07)	− 0.53*** (0.07)	− 0.47*** (0.08)	− 0.47*** (0.08)
Legal profession		− 0.10 (0.13)	− 0.13 (0.10)	− 0.02 (0.13)	− 0.02 (0.13)
Analysis # Legal profession				− 0.06 (0.15)	− 0.06 (0.15)
Selection # Legal profession				− 0.08 (0.13)	− 0.08 (0.13)
Implementation # Legal profession				− 0.29** (0.15)	− 0.29** (0.15)
R-squared	0.05	0.07	0.26	0.26	0.26
Demographics	N	Y	Y	Y	N
Additional controls	N	Y	Y	Y	N
Respondent FE	N	N	N	N	Y
Number of observations	1067	984	984	984	984

This table presents OLS results. The dependent variable is the LOA perceived as most fair by the respondent. Demographics are: Female, Age, Age-squared, Legal Profession, and Court Experience. Additional Controls are Knowledge in Tech, Trust in Tech, Personal Innovativeness, and Compatibility. The reference category for the stage is *information acquisition*. Standard errors are clustered by respondent. *p < 0.1 **p < 0.05 ***p < 0.01

Table 3 reveals several key insights. *First*, the coefficients of LOA in the later stages of the judicial decision-making process (analysis, selection, implementation) are all negatively significant (*p* < 0.001). This suggests that the initial stage of *information acquisition* is believed to generate fairer results with a higher level of automation. In other words, individuals believe that the use of technology in later stages of the process is more likely to lead to unfair outcomes.

*Second*, respondents with a legal profession only differ from others in their perception of using automation in the implementation stage, with a negative coefficient for the interaction term (− 0.29, *p* < 0.005), suggesting they perceive automated implementation as relatively unfair.

*Third*, when comparing the coefficients of the three stages listed in the table (analysis, selection, and implementation), the coefficients are of similar size (and in fact, they are not significantly different from one another, as confirmed by a Wald test). This reaffirms that the results are driven by a distinction between the information acquisition and other stages (and not the differences across the three other stages).

## Conclusion

Our study of beliefs about the level of automation in different stages of the judicial decision-making process reveals several interesting findings.

Firstly, people seem to believe that low levels of automation would ensure the fairest outcomes in judicial decision-making. The intermediate level of automation was preferred only in the first-information acquisition-stage. These results are consistent with the emerging literature on perceived algorithmic fairness within the law, which indicates that people might generally trust judges more than algorithms (Hermstrüwer and Langenbach 2022; Yalcin et al. 2022). However, it also suggests that a binary view of judges vs. algorithms might be insufficient for capturing how people perceive automation in the court, as perceptions change depending on the stage of the decision-making process. This finding might also be explained by the more general distinctions on trust in AI, as captured by the aforementioned paper by Glikson and Woolley (2020). Namely, ADM used in courts neither manifests itself to the public in a tangible way nor is it transparent, both of which tend to lead to lower trust.

Secondly, the evidence suggests that automation is perceived as most likely to generate fair outcomes in the first decision-making stage (information acquisition), which seems counter-intuitive: usually, one might expect individuals to trust algorithms in the analysis of the information more than its acquisition.^37^ At the same time, people might be simply more familiar with the concepts of automatic information retrieval due to e.g., their day-to-day use of online search engines. As search engines have arguably gotten better over time, individuals may anticipate a similar process in ADM, which increases trust in information acquisition. However, other explanations for our finding may be found by turning to concepts from behavioral law and economics. For instance, suppose that judges fall pray to the so-called “confirmation bias” (see, e.g., Jones and Sugden 2001; for experimental evidence on adjudication, see Eerland and Rassin 2012), where they first form an opinion and then collect only the information that is consistent with that opinion. Individuals who anticipate the bias might then prefer to let an algorithm collect the evidence. Moreover, the presence of biases in the first stage might spillover to the following stages, e.g. because judges may turn to heuristics already from the beginning and this will form the basis for the subsequent stages.^38^ An alternative explanation would be distrust in the current algorithm’s ability to perform analysis, selection and implementation, e.g., due to the usual aforementioned concerns of fairness and potential bias of ADM (for a discussion, see Kim 2022). In particular, implementation, unlike analysis, might be perceived as an inherently human process, requiring capabilities that are simply irreplaceable by a computer (see, e.g., Kasy and Abebe 2021). This is particularly true if implementation involves emotions (Yalcin et al. 2022; Xu 2022; Ranchordas 2022), e.g., allowing a human judge to incorporate equity concerns or compassion. Recall, however, that individuals with a legal profession in our study have an even stronger belief that automation in implementation is likely to yield unfair outcomes. This might be driven by either a true belief (e.g., due to conservatism, or to personal experience in representing clients in front of human judges) or political economy: if lawyers believe that their added value is in influencing implementation (e.g., by submitting written arguments to the judge before verdicts are written), they might object to automation in order to protect their stream of income.

Although our study is exploratory, the findings may potentially hold several important policy implications. First, our finding that the perceived fairness of automation is higher in the information collection stage implies that judges who are interested in maintaining their public support might take more liberty in using technology in the earlier stages of the process but avoid technology in later stages. From the perspective of judicial administration, one might even consider actively restricting judges from using automation for some actions, if judges prefer to save on effort costs and neglect the cost of lowering public support. Second, the stronger perception of lawyers regarding the unfairness of automated implementation may be especially important, as discontentment with automation might lead lawyers to communicate their criticism of the court to the clients. In other words, one might need to assign more weight to the preferences of lawyers because they might spill over to the clients.

Thirdly, the different stages we consider might be more relevant in inquisitorial systems—where the judge actively collects data—than in adversarial systems. The same is true for appeal systems in civil law systems, in which new evidence can be collected more easily in the appeal compared to common law systems (see, e.g., Feess and Sarel 2018). Of course, one should take this distinction with a grain of salt, as there may be second-order effects (e.g., if judges use automation to collect data, litigants may anticipate this and respond by hiding some information).

Lastly, we asked respondents to specify what they believe would yield a fairer outcome, i.e. we elicited their *beliefs* about fairness. This means that we do not directly ask whether they also *prefer* to have a fair process. As it is hard to imagine that people dislike fairness, it is plausible that respondents who believe that a certain process will yield a fair outcome also prefer to have that process in place. Therefore, our findings may well reflect the public’s preferences and not merely beliefs. Nonetheless, further research is needed to clarify this point, as it is possible that some specific sub-groups actually prefer unfair outcomes (e.g. guilty criminal defendants who would rather be unfairly exonerated).

Our study is subject to a few limitations. First, it is an exploratory study and, as such, uses simplified questions that aim at general opinions toward automation rather than specific opinions regarding adjudication fields. Nonetheless, it seems sufficient to illustrate the general point that differences exist between perceptions of technology at different stages. We leave the exploration of differences between legal fields for future studies. Second, our study builds on the existing literature, the highly-cited paper by Parasuraman et al. (2000), and applies discrete levels of automation, which has the advantage of keeping things simple for the subjects. However, future studies may well benefit by considering a more intricate distinction, such as the one proposed by Tamò-Larrieux et al. (2022). Third, as our pool of participants includes also non-professionals, one may question whether their views are essential. However, attitudes of the general public about court processes are no less critical to court legitimacy than experts’ opinions. Fifth, our use of Lithuanian respondents means that we can only capture opinions made against the background of judicial processes in Lithuania. A cross-country follow-up study may help establish whether our results generalize to other countries (in particular, given the possibility that inquisitorial systems differ from adversarial ones). Sixth, as discussed above, our convenience sampling is subject to limitations as well. Finally, our choice to restrict attention to fairly general questions might overlook the fine-grained details of the technology. Consequently, a different design, in which further details are provided on the technology’s capacity or functioning might yield different results. Future work would benefit from such attempts and shed further light on the ever-evolving issues discussed in this paper.

## Appendix A

See Tables

**Table 4:** Bivariate Correlations

Variables	(1)	(2)	(3)	(4)	(5)	(6)	(7)	(8)
(1) Age	1.000							
(2) Female	0.157***	1.000						
	(0.000)							
(3) Court experience	0.162***	− 0.059*	1.000					
	(0.000)	(0.058)						
(4) Legal profession	− 0.201***	− 0.163***	0.375***	1.000				
	(0.000)	(0.000)	(0.000)					
(5) Trust in legal tech	− 0.173***	− 0.028	− 0.082***	− 0.053*	1.000			
	(0.000)	(0.371)	(0.008)	(0.081)				
(6) Relative advantage	− 0.180***	0.102***	− 0.083***	− 0.075**	0.810***	1.000		
	(0.000)	(0.001)	(0.007)	(0.015)	(0.000)			
(7) Personal innov	− 0.267***	− 0.064**	0.089***	0.097***	0.333***	0.377***	1.000	
	(0.000)	(0.037)	(0.003)	(0.002)	(0.000)	(0.000)		
(8) Compatibility	− 0.178***	0.049	0.000	0.029	0.825***	0.859***	0.393***	1.000
	(0.000)	(0.111)	(0.988)	(0.344)	(0.000)	(0.000)	(0.000)	

This table presents bivariate (pairwise) correlations between the independent variables used in the analysis. *** *p* < 0.01, ** *p* < 0.05, * *p* < 0.1

^4^,

**Table 5:** Descriptive statistics by legal profession

Factor	Non-legal profession	Legal profession	*P* value	Test
Number of observations	213	53		
Age, mean (SD)	42.6 (12.64)	36.44 (10.12)	0.001	Two sample t-test
Female	136 (65.1%)	23 (45.1%)	0.009	Pearson’s $\chi^{2}$
Court experience	93 (43.7%)	48 (90.6%)	< 0.001	Wilcoxon Rank-sum
Knowledge in Legal Tech, median (IQR)	1.43 (1, 2)	2 (1.57, 2.71)	< 0.011	Wilcoxon Rank-sum
Trust in Legal Tech, median (IQR)	5 (4, 5.66)	5 (3.33, 5.66)	0.49	Wilcoxon Rank-sum
Relative advantage, median (IQR)	5.25 (4.4,6)	5 (4,6)	0.22	Wilcoxon Rank-sum
Personal Innov., median (IQR)	4.75 (4,5.75)	5 (4,6.25)	0.16	Wilcoxon Rank-sum
Compatibility, median (IQR)	5 (4, 5.75)	5.25 (4.5, 5.75)	0.39	Wilcoxon Rank-sum

This table presents descriptive statistics by legal profession. The third column presents the p-value generated from the test specified on the fourth column when comparing respondents with and without a legal profession

5,

**Table 6:** Percentage of LOAs chosen by Stage

Level of automation	Judicial decision-making stage			
	Information acquisition	Information analysis	Decision selection	Decision implementation
Manual	26 (9.7%)	38 (14.1%)	42 (15.6%)	48 (17.8%)
Low	54 (20.1%)	131 (48.7%)	131 (48.7%)	123 (45.7%)
Intermediate	106 (39.4%)	52 (19.3%)	57 (21.2%)	45 (16.7%)
High	73 (27.1%)	43 (16.0%)	36 (13.4%)	45 (16.7%)
Full	5 (1.9%)	3 (1.1%)	3 (1.1%)	6 (2.2%)
Stage is irrelevant	5 (1.9%)	2 (0.7%)	0 (0.0%)	2 (0.7%)

This table presents the distribution of respondents who choose each level of automation depending on the stage in the judicial process. The information is also available graphically as Fig. 1 in the main text

6,

**Table 7:** Comparison of Perceived fairness of LOAs by different characteristics

*Panel A: Legal profession Court Experience*	Information Acquisition, median (IQR)	Information Analysis, median (IQR)	Decision Selection, median (IQR)	Decision Implementation, median (IQR)
Non-legal profession (N = 206)	3 (2,4)	2 (2,3)	2 (2,3)	2 (2,3)
Legal profession (N = 53)	3 (3,4)	2 (2,3)	2 (2,3)	2 (2,3)
No court experience (N = 121)	3 (2,4)	2 (2,3)	2 (2,3)	2 (2,3)
Court experience (N = 140)	3 (2,4)	2 (2,3)	2 (2,3)	2 (2,3)

*Panel B: Age and gender*				
Age < 40 (N = 131)	3 (3,4)***	2 (2,3)	2** (2,3)	2 (2,3)
Age > = 40 (N = 138)	3 (2,3)	2 (2,3)	2 (2,3)	2 (2,3)
Male (N = 102)	2 (2,4)	2 (2,4)	2 (2,3)	2 (1,3)
Female (N = 153)	3 (2,4)	2 (2,3)	2 (2,3)	2 (2,3)

This table compares the median perceived fairness across characteristics. Respondent who identified any stage as irrelevant are excluded. Between-group comparison are conducted using Wilcoxon Ranksum. Significantly higher values are marked as follows: **p* < 0.1 ***p* < 0.05 ****p* < 0.01

^7^ and

**Table 8:** Full OLS results

	(1)	(2)	(3)	(4)	(5)
Analysis	− 0.50*** (0.06)	− 0.49*** (0.07)	− 0.49*** (0.07)	− 0.48*** (0.08)	− 0.48*** (0.08)
Selection	− 0.56*** (0.06)	− 0.54*** (0.07)	− 0.54*** (0.07)	− 0.52*** (0.08)	− 0.52*** (0.08)
Implementation	− 0.52*** (0.07)	− 0.53*** (0.07)	− 0.53*** (0.07)	− 0.47*** (0.08)	− 0.47*** (0.08)
Age		− 0.02 (0.02)	− 0.02 (0.02)	− 0.02 (0.02)	− 0.02 (0.02)
Age # Age		0.00 (0.00)	0.00 (0.00)	0.00 (0.00)	0.00 (0.00)
Female		− 0.08 (0.11)	− 0.10 (0.09)	− 0.10 (0.09)	− 0.10 (0.09)
Legal profession		− 0.10 (0.13)	− 0.13 (0.10)	− 0.02 (0.13)	− 0.02 (0.13)
Court experience		0.04 (0.10)	0.02 (0.09)	0.02 (0.09)	0.02 (0.09)
Knowledge in legal tech			0.21*** (0.07)	0.21*** (0.07)	0.21*** (0.07)
Trust in legal tech			0.19*** (0.06)	0.19*** (0.06)	0.19*** (0.06)
Legal tech compatibility			− 0.01 (0.06)	− 0.01 (0.06)	− 0.01 (0.06)
Relative advantage			0.11* (0.07)	0.11* (0.07)	0.11* (0.07)
Personal innovativeness			− 0.01 (0.03)	− 0.01 (0.03)	− 0.01 (0.03)
Analysis # Legal profession				− 0.06 (0.15)	− 0.06 (0.15)
Selection # Legal profession				− 0.08 (0.13)	− 0.08 (0.13)
Implementation # Legal profession				− 0.29** (0.15)	− 0.29** (0.15)
Constant	2.91*** (0.06)	3.54*** (0.53)	1.78*** (0.46)	1.76*** (0.46)	1.76*** (0.46)
R-squared	0.05	0.07	0.26	0.26	0.26
Number of observations	1067	984	984	984	984

This table presents the full OLS results for the regression specified in Table 2

^8^.

## Appendix B

See Tables

**Table 9:** Items of the survey

Construct	Revised/adopted from	English version
Trust in legal technologies	Min et al. (2019), Moore and Benbasat (1991), Wang et al. (2018), Yuen et al. (2018)	I think legal technologies would help make fair decisions in court processes
		I think it would be entirely safe to entrust legal technologies with some parts of the litigation (e.g., accepting documents and analyzing arguments for the ruling)
		Overall, I could trust legal technologies in courts
Relative advantage	Lee (2018), Vimalkumar et al. (2021), Zhang et al. (2020)	I think legal technologies would make it more convenient to perform specific court processes compared to how courts operate now (e.g., document submission, resolution of some disputes, argumentation of the verdict)
		I think legal technologies would help save time for court clients and staff compared to how courts operate now
		In my opinion, compared to how courts operate now, legal technologies would ensure fewer mistakes are made in court processes
		I think, compared to how courts operate now, legal technologies would ensure greater control over specific court processes and their results (e.g., there would be more transparency)
		Compared to how courts operate now, I think legal technologies would improve the overall court experience for court clients and staff
Compatibility	Agag and El-Masry (2016), Heydari et al. (2020), Min et al. (2019), Moore and Benbasat (1991), Wang et al. (2018)	I think legal technologies would be well in line with my beliefs about how courts should operate
		As far as I would have to deal with courts, using legal technologies in courts would be compatible with how I like to address issues in my life
		I think legal technologies would be well compatible with my needs in court, as far as I would have to deal with them (e.g., for the court client—to defend their interests, for the employee—to perform their work effectively)
		In my opinion, implementing legal technologies in courts would be compatible with current trends in the extent to which various areas of life are automated
Personal Innovativeness in information technology	Lu et al. (2005), Patil et al. (2020)	If I heard about new relevant information technology, I would look for ways to experiment with it
		I am usually the first to explore new information technologies among my peers
		I like to experiment with new information technologies
		In general, I am hesitant to try out new information technologies

This table presents the statements shown to respondents in the survey and the references for papers from which the statements were adapted

9 and

**Table 10:** Descriptions of judicial decision-making and LOA’s used in the survey

Decision making stage	Description
Information acquisition	When deciding a case, a judge needs a wide range of information to decide a case—legislation, decisions in similar cases, and legal arguments. The alternatives for outcome and arguments depend on the information found
	Please read the following options and choose which option you think would best provide a final solution in most cases:
	[Automation level 0] The Judge is the only source for searching, filtering and selecting information
	[Level 1] Searching and filtering information is done in the program using keywords, and the judge must select the most relevant information
	[Level 2] Searching and filtering information is done by entering keywords in the program, the program highlights the most relevant information, but the judge must select the most relevant information
	[Level 3] Information search and filtering is done in the program according to the keywords selected by the program, the program selects the most relevant information, and everything is displayed to the judge
	[Level 4] The program performs a full search for the required information, the judge is not presented with the search results
	[-] This decision-making stage is not relevant to ensure a fair final decision in the case
Information analysis	The collected information is analyzed and interpreted to choose the best solution. The judge can investigate relations among arguments, the relative weight of the arguments, and the reasonableness of the possible decisions for the case
	Please read the following options and choose which option you think would best provide a final solution in most cases (all levels of automation are available regardless of the previous level of automation you would have chosen):
	[Automation level 0] The judge does all of the analysis and interpretation needed
	[Level 1] The program performs the analysis, but it is up to the judge to interpret and relate the analysis to the case outcomes
	[Level 2] The program performs analysis and interpretation; the judges can read the analysis and interpretation, adjust only if necessary
	[Level 3] The program performs analysis and interpretation; the judge can only read through the analysis and interpretation
	[Level 4] The program performs the analysis and interpretation; no information is provided to the judge
	[-] This decision-making stage is not relevant to ensure a fair final decision in the case
Decision selection	In the decision selection stage, the judge can rate the available alternatives (possible options for the final decision and arguments) according to which alternative would be the most appropriate
	Please read the following options and choose which option you think would best provide a final solution in most cases (all levels of automation are available regardless of the previous level of automation you would have chosen):
	[Automation level 0] The judge assesses which decision alternatives and arguments are the most appropriate, and which are less appropriate
	[Level 1] The program and the judge evaluate alternatives; the judge may consider the program proposals
	[Level 2] The program evaluates alternatives, and the judge is provided with explanations to support the evaluations. The judge will evaluate the alternatives only if necessary
	[Level 3] The program evaluates alternatives, and the judge is provided with explanations to support the evaluations. The judge has access to a reduced list of the most suitable alternatives; no explanations are given
	[Level 4] The program selects the most appropriate arguments without explanation. The judge is not present at this stage of the decision-making process and does not receive any information
	[-] This decision-making stage is not relevant to ensure a fair final decision in the case
Decision implementation	In the last stage, the most suitable solution is implemented
	Please read the following options and choose which option you think would best provide a final solution in most cases (all levels of automation are available regardless of the previous level of automation you would have chosen):
	[Automation level 0] Only the judge can prepare a court document with the final decision and its reasoning
	[Level 1] The program prepares a text with a final solution and reasoning. The judge can accept the text, edit it, prepare his/her own version
	[Level 2] The program prepares a text with a final solution and reasoning. The judge can reject the prepared text within a certain period of time. In exceptional cases, the judge prepares the text herself/himself
	[Level 3] The program prepares a text with a final solution and reasoning. The judge can familiarize himself with the prepared document. In exceptional cases, the judge may edit the decision
	[Level 4] The program makes a decision and prepares a text with reasoning. At this decision-making stage, the judge does not participate, does not receive any information, and cannot change the decision
	[-] This decision-making stage is not relevant to ensure a fair final decision in the case

This table presents the text used to explain the LOAs to the respondents in the survey

10.

## Footnotes

1 In addition to judicial tools, AI is indirectly involved in litigation through software used by litigants, e.g. automated document searches, prognosis of case outcomes (Kluttz and Mulligan 2019; Suarez 2020), and other tools that try to substitute for lawyers (Sandefur 2019; Sourdin and Li 2019; Janeček et al. 2021).

2 Newell and Marbelli (2015, p. 4) describe ADM as the case where “data are collected through digitized devices carried by individuals such as smartphones and technologies with inbuilt sensors—and subsequently processed by algorithms, which are then used to make (data-driven) decisions”. Note that some papers replace the word “Algorithmic” with “Automated”, yielding the same acronym of “ADM”.

3 Reichman et al. (2020) discuss the development of a technological system in Israel (“Legal-Net”), identifying both advantages for judges (e.g. it became easier to track the case’s progress) and disadvantages (e.g. it potentially created incentives to “conform” by issuing quick decisions at the expense of other considerations). Winmill (2020) describes how the use of technology in an Idaho court substantially enhanced efficiency.

4 For a similar argument regarding lawyers, see Alarie et al. (2018).

5 There are other critiques of using AI in legal decision-making. e.g., that certain types of AI over-rely on the assumption that past data can be used to predict the future (Fagan and Levmore 2019), that AI cannot properly judge whether the burden of proof is met (see Aini 2020), that AI outputs might be incomprehensible or alienated (Re and Solow-Niederman 2019), or that AI will unlikely produce high-quality decisions when data is insufficiently granular (Xu 2022).

6 However, there is also recent experimental evidence suggesting that judges may be less susceptible to framing effects than law students and laypeople (van Aaken and Sarel 2022).

7 Race was not an explicit element of the COMPAS software. Instead, the outcomes were discriminatory because of statistical discrimination (e.g., Conklin and Wu 2022; Hübner 2021).

8 Glikson and Woolley (2020) also differentiate between “cognitive trust”—a rational evaluation of the trustee—and “emotional trust”—a form of trust driven by feelings, affect, or personal connections.

9 Trust may also be affected by the degree of awareness to the technology, as many people may be under-informed about how technology is used, e.g. because they lack expertise. Still, even without perfect knowledge, people may hold opinions and preferences over the usefulness of—and need for—technologies in the courts (see, e.g., Barysė 2022a, b).

10 There are, however, various theoretical arguments on the optimal mix of automation and human-judging, mostly concluding that one should delegate some (but not all) functions to AI (Fagan and Levmore 2019; Ulenaers 2020; Cofone 2021).

11 We discuss some possible explanations for why lawyers’ perceptions may differ in Sect. 5.

12 https://techindex.law.stanford.edu/.

13 Namely, Marketplace, Document Automation, Practice Management, Legal Research, Legal Education, Online Dispute Resolution, E-Discovery, Analytics, and Compliance.

14 Legal directness refers to whether the technology engages directly with the law, whereas legal specificity asks whether the technology is generic or specifically applied only to legal issues.

15 The two papers by Guitton et al. use slightly different terms but are conceptually similar. The first dimension, Aim (or “primary benefit”) refers to whether the technology is meant to improve the accessibility to regulation or rather its efficiency.Divergence of interests concerns those sponsoring the technology, those implementing it, and its users. Human mediation concerns the degree of human (dis)integration, considering factors such as domain, code, and data.

16 Tamò-Larrieux et al. (2022) discern different types of autonomy, namely: autonomy in the choice of goal, autonomy in its execution, and autonomy from the environment, other agents, and organizations.

17 Parasuraman et al. (2000) is an influential paper (with over 4000 citations as of August 29, 2022 according to Google Scholar) and has been applied to conceptualize various decision-making processes. We build on this paper, as well as a working paper by (Petkevičiūtė-Barysienė 2021), which applies the framework also to a legal context..

18 Proud et al. (2003) use an 8-point scale, but we simplified by it switching to a 5-point scale (as in, e.g., Petkevičiūtė-Barysienė, 2021).

19 Woodruff et al. (2018) studies qualitatively how knowledge on algorithmic fairness affects individuals’ perception of firms who use algorithms, finding a negative affect of unfairness on trust in those firms. Lee (2018) finds that algorithmic decisions are perceived as less fair when the task typically requires human skills. Lee et al. (2019) contrast different features of algorithmic fairness and find higher perceived fairness in response to some features of transparency (e.g., when the outcome of the algorithm was explained) but not others. Saxena et al. (2019) finds that algorithms that express “calibrated fairness”, which is based on meritocracy, are perceived as relatively fair.

20 Note that our study does not ask respondents directly about procedural fairness. Instead, it elicits beliefs about the LOA that is most likely to ensure the fairest outcomes. As such, the study is asking people to construe a fair process.

21 Examples of other factors include complexity (how difficult the innovation is to understand or use), trialability (the extent to which the innovation can be tested before adoption), and observability (the extent to which the innovation provides tangible results).

22 The survey was conducted in the Lithuanian language, but is presented henceforth using an English translation.

23 https://e.teismas.lt/en/public/services/.

24 https://www.teismai.lt/en/national-courts-administration/activities/competence-areas/685.

25 For another study using survey evidence from Lithuania in the context of technologies, see Kumpikaitė and Čiarnienė (2008). For a discussion of the AI strategy in Lithuania in general, see Daugeliene and Levinskiene (2022).

26 Teise.pro.

27 Vilnius University, Mykolas Romeris University.

28 E.g., “Apklausos” (“Surveys”), “Tyrėjai-tiriamieji” (“Researchers-respondents”).

29 https://www.mctau.lt/apie-mctau/tikslai.

30 Cronbach’s alpha, which is a test used to verify that the statements are indeed related, is as follows: Knowledge in Tech–0.897; Trust–0.872; Relative Advantage–0.911; Compatibility–0.927; Personal innovativeness 0.842. This suggests that the statements in each set are indeed related.

31 The questions for this point were restricted to knowledge about what kind of technologies for courts exist, without discussing how they work or any specific details. Our choice to use a general question is related to the issue underlying our study—that the existing attempts to classify AI lead to imperfect results We hence thought it is best to simply captures one’s general acquaintance with the technology, without going into details. While this yields a tradeoff, as we might miss the subtleties of precisely how one uses the technology, it seems preferable for two reasons. First, our sample does include legal professionals, so that there is a possibility that they encountered the technology directly (yielding a need to control for such acquaintance). Second, some aspects of the technology could, in principle, be used by individuals (e.g. if any collection of the evidence is done electronically).

32 Recall that Personal Innovativeness might be determined by the relative advantage and compatibility, but also by other factors. Hence, we controlled for this separately, to ensure that all the features are covered by our controls.

33 We also included an option of “This stage of the decision is not relevant to ensure a fair final decision in the case”. However, only a few people chose this option (see Table 6 for descriptive statistics).

34 Participants were asked to indicate whether they had court experience and name their role in the process, e.g., judge, litigant, attorney, observer.

35 We excluded from the graph the few respondents who indicated that the stage is irrelevant. Details on the amount of these respondents are available in Table 6 in Appendix A.

36 In columns (2)–(5) there are missing observations for some controls, as we permitted subjects not to provide information for some questions.

37 In particular, the analysis of the information is probably viewed as more technical task, which generally has been found to increase trust (Glikson and Woolley 2020).

38 This is consistent with the well-established idea of a dual-process system (see, e.g., Bago and De Neys 2020), where individuals first use heuristics (“System I”) and only then turn to deliberate thinking (“System II”).
